# Computational Studies of Benzoxazinone Derivatives as Antiviral Agents against Herpes Virus Type 1 Protease

**DOI:** 10.3390/molecules200610689

**Published:** 2015-06-10

**Authors:** Juliana F. R. e Mello, Nathália C. Botelho, Alessandra M. T. de Souza, Riethe de Oliveira, Monique A. de Brito, Bárbara de A. Abrahim-Vieira, Ana Carolina R. Sodero, Helena C. Castro, Lucio M. Cabral, Leonardo A. Miceli, Carlos R. Rodrigues

**Affiliations:** 1 Laboratory of Molecular Modeling & QSAR Faculty of Pharmacy, Federal University of Rio de Janeiro, Rio de Janeiro 21.944-970, RJ, Brazil; E-Mails: nathbc1991@hotmail.com (N.C.B.); amtsouza2@yahoo.com.br (A.M.T.S.); ri_oliver@msn.com (R.O.); babi_abrahim@hotmail.com (B.A.A.-V.); acrsodero@gmail.com (A.C.R.S.); 2 Laboratory of Computational Medicinal Chemistry, Faculty of Pharmacy, Fluminense Federal University, Niterói 24.241-000, RJ, Brazil; E-Mail: moniquebrito@id.uff.br; 3 Laboratory of Antibiotics, Biochemistry, Education and Molecular Modeling, Institute of Biology, Fluminense Federal University, Niterói 24.210-130, RJ, Brazil; E-Mails: hcastrorangel@yahoo.com.br (H.C.C.); leossj@hotmail.com (L.A.M.); 4 Laboratory of Industrial Pharmaceutical Technology, Faculty of Pharmacy, Federal University of Rio de Janeiro, Rio de Janeiro 21.944-970, RJ, Brazil; E-Mail: lmcabral2@ig.com.br

**Keywords:** herpesvirus, protease, molecular docking, comparative modeling, benzoxazinones

## Abstract

Herpes simplex virus infections have been described in the medical literature for centuries, yet the the drugs available nowadays for therapy are largely ineffective and low oral bioavailability plays an important role on the inefficacy of the treatments. Additionally, the details of the inhibition of Herpes Virus type 1 are still not fully understood. Studies have shown that several viruses encode one or more proteases required for the production new infectious virions. This study presents an analysis of the interactions between HSV-1 protease and benzoxazinone derivatives through a combination of structure-activity relationships, comparative modeling and molecular docking studies. The structure activity relationship results showed an important contribution of hydrophobic and polarizable groups and limitations for bulky groups in specific positions. Two Herpes Virus type 1 protease models were constructed and compared to achieve the best model which was obtained by MODELLER. Molecular docking results pointed to an important interaction between the most potent benzoxazinone derivative and Ser129, consistent with previous mechanistic data. Moreover, we also observed hydrophobic interactions that may play an important role in the stabilization of inhibitors in the active site. Finally, we performed druglikeness and drugscore studies of the most potent derivatives and the drugs currently used against Herpes virus.

## 1. Introduction

The Herpesviridae family is one of the major viral families. It includes 100 identified viruses that affect almost all animal species [1]. The infections caused by Herpes simplex virus type 1 (HSV-1) are prevalent worldwide and have been described in the medical literature for centuries, but they remain incurable [2,3]. Clinical manifestations of HSV infections can range from asymptomatic or symptomatic versions (e.g., oral, labial, mucocutaneous, ocular, genital herpes, eczema herpeticum) to central neurological complications such as neonatal herpes, herpes encephalitis and even a fatal dissemination, mainly seen in immunocompromised patients [4,5].

Nowadays, several drugs are used in HSV therapy [6,7,8]. Although the current therapy against these viruses presents a safe profile, the low oral bioavailability plays an important role in compromising the treatment efficacy, therefore, the discovery of new potent and safe molecules with high oral bioavailability is of considerable interest.

In the last 20 years, the literature has described that several viruses that codify one or more proteases involved in protein catalysis or the capsid maturation process, a crucial process for virion production [9,10]. One example is the HSV-1 serine protease, which participates in the viral capsid organization process. This serine protease has emerged as an important therapeutic target for the development of new antiviral agents due to its structural features such as non-homologous folding [11,12,13,14]. Several serine protease inhibitors of herpes viruses have been reported in the recent years. In general, the mechanism of the protease inhibitors involves the classical reaction with the serine residue of the active site (Ser-His-His catalytic triad) thus inactivating the HSV enzyme [15].

The benzoxazinones were identified in 1960 as secondary metabolites of grasses and currently are known as serine protease inhibitors [16,17,18]. The inhibitory mechanism of benzoxazinones against HSV-1 protease was proposed by Jarvest and coworkers [18], who suggested the formation of an acyl-enzyme complex with Ser129 from the catalytic triad. Further evidences also pointed to interactions with the residues His61, His148, Arg156 and Arg157 as important for HSV-1 protease inhibition [11,15,18,19].

Advances in computer technology have driven the use of *in silico* modeling techniques, including molecular docking, in drug discovery and development research [11]. This technique can be very helpful to discover new molecules since it enables the study of intermolecular complexes. In cases where the tridimensional structure of the target is not available, comparative modeling can be used as a tool to predict the tridimensional structure of the protein/enzyme before conducting the docking evaluation [20]. Other parameters such as druglikeness and drugscore calculations may also be used to rationalize how the physicochemical properties may influence *in vivo* studies [21].

In order to study the active site profile and the binding mode of HSV-1 protease with a series of potential inhibitors and to propose a lead compound, herein we present the structure-activity relationship (SAR), comparative modeling, molecular docking and in *silico* pharmacokinetic studies of a series of benzoxazinones [18].

## 2. Results and Discussion

### 2.1. Structure-Activity Relationship

In order to determine the structural and stereoelectronic features that could determine the anti HSV-1 protease profile, first we evaluated the structure-activity relationships (SAR) of the studied benzoxazinone series. The activities were previously obtained by Jarvest and co-workers [18]. The comparison of the two groups (oxy- and aminobenzoxazinones) showed better antiviral activity for the oxybenzoxazinones.

This biological activity can be associated to the electronegativity of the oxygen compared to the nitrogen atom (Table 1) and the capacity to be only a hydrogen bond acceptor. The structure-activity relationship analysis of this series suggested that bulk, lipophilic and aliphatic substituents on the R^1^ position decreased or even abolished (compound **9**) the inhibitory activity. Besides, deactivating groups linked to a phenyl in R^1^ could also decrease the activity (compound **7**), highlighting a high electronic density as a requirement in this region.

Analysis of the R^2^ position, specially comparing asparagine (Asn) and alanine (Ala) residues, revealed Asn as deleterious for activity for both amino- and oxybenzoxazinone compounds (**4**, **5**, **13** and **14**, Table 1). In contrast, substitution of an Ala residue at R^2^ position increases the activity, which can be related to its hydrophobic feature and volume.

The occurrence of hydrophobic interactions should be considered for these series since this type of interactions is related to atom polarizability and lipophilicity and may be important for the activity. Lipophilic and polarizable groups located on the R^3^ position seem to be good for the inhibitory activity, as observed in compounds **13** and **15**. The same influence is observed for substitutions at R^4^ position as shown by compound **15** (R^4^ = Cl). Interestingly, these studies revealed some important structural features of these molecules apparently related to the activity (Table 1). The most active molecules presented molecular weights and the volumes ranging from 269.2 to 445.9 amu and 261.9 to 424.4 Å^3^, respectively. Molecules showing values above or below the highlighted ranges were less active (Table 1). In contrast, the analysis of the calculated electronic parameters of these compounds (such as EHOMO, ELUMO and dipole moment) and the correlation with activities did not show any direct relationships (data not shown).

**Table 1:** Comparison between *in vitro* activity of benzoxazinones **1**–**18** and the theoretical calculated parameters including Molecular Weight (MW), Molecular Volume (MV) and lipophilicity (cLog P). 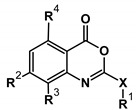

Compound	X	R^1^	R^2^	R^3^	R^4^	IC_50_ (µM)	MW (amu)	MV (Å^3^)	cLog P
**1**	N	Bu	H	H	H	300.0	218.26	228.46	3.10
**2**	N	iPr	H	H	H	25.0	204.23	210.10	2.52
**3**	N	iPr	NH_2_	H	H	55.0	219.24	220.39	1.71
**4**	N	iPr	Cbz-Ala-NH	H	H	5.0	424.46	424.42	3.09
**5**	N	iPr	Cbz-Asn-NH	H	H	15.0	467.48	453.58	1.66
**6**	N	iPr	iBuCOONH	H	H	50.0	319.36	325.45	3.23
**7**	N	MeO_2_CCMeH	H	H	H	25.0	248.24	240.08	1.88
**8**	N	(R)-PhCMeH	H	H	H	60.0	266.30	275.00	3.91
**9**	N	3-F-Ph	H	H	H	>300	256.24	242.65	3.68
**10**	O	Et	H	H	H	25.0	191.19	188.12	2.58
**11**	O	Et	H	H	F	75.0	209.18	192.67	2.73
**12**	O	Et	NH_2_	H	Me	15.0	220.23	215.78	2.26
**13**	O	Et	Cbz-Ala-NH	H	Cl	7.0	445.86	415.55	3.71
**14**	O	Et	Cbz-Asn-NH	H	Et	20.0	482.49	469.31	2.62
**15**	O	Et	iBuCOONH	Cl	Cl	1.5	375.21	328.83	4.41
**16**	O	Allyl	H	H	H	15.0	203.20	202.15	2.93
**17**	O	Ph	H	H	H	10.0	239.23	234.89	3.90
**18**	O	4-OCH_3_-Ph	H	H	H	2.5	269.26	261.92	3.78

### 2.2. Comparative Modeling

**Figure 1:**
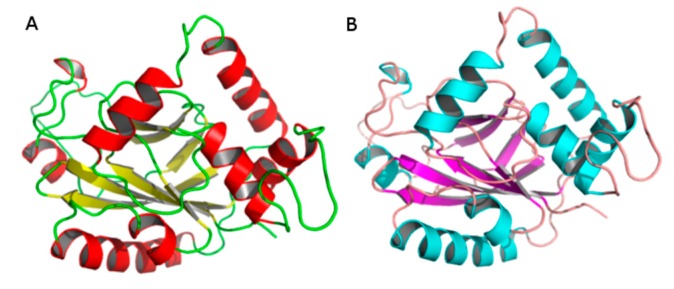
Models of HSV-1 protease generated by SWISS-MODEL and Modeller softwares. (**A**) Model obtained with Modeller software and (**B**) Model obtained with SWISS-MODEL software.

The crystal structure of HSV-2 protease (PDB ID: 1AT3) was selected as potential template as the result of a BLAST-P search. The three-dimensional structure of HSV-1 protease was constructed employing SWISSMODEL and Modeller (Figure 1), as previously described. Stereochemical validation of the models structures is an important step of comparative modeling, used to evaluate the accuracy of the 3D-structure representation.

The Ramachandran Plot exhibited 90.8% of residues of the model obtained with Modeller localized in most favored regions and none in disallowed regions. Meanwhile the model generated from SWISS-MODEL showed 83.7% of residues localized in most favored regions and 1.5% in disallowed regions. Verify3D results showed 87.9% and 81.1% of the amino acids in the Modeller and SWISS-MODEL models have compatible 1D-3D scores greater than 0.2 (Table 2) suggesting that both models have overall self-consistency in terms of sequence-structure compatibility.

**Table 2:** Results of validation step Procheck and Verify 3D for the two models constructed by using Modeller and SWISS-MODEL programs compared to two models obtained from the literature.

Crystal Structure	Modeller	SWISS-MODEL
**Ramachandran plot ^a^ (%)**	90.80	83.7
**Ramachandran plot ^b^ (%)**	0	1.5
**Verify 3D ^c^ (%)**	87.93	81.3

^a^ Residues on most favored regions. ^b^ Residues in disallowed regions. ^c^ Average 3D-1D profile score for each residues in a 21-residue sliding window.

Thus, according to our validation data (Table 2), the best model was obtained with the Modeller program. Both models presented quite separated catalytic residues, allowing the entry and cleavage of a polypeptide substrate at the active site.

### 2.3. Molecular Docking

#### 2.3.1. Docking Validation

The validation of the docking protocol was carried out through a re-docking using the crystal structure of Cytomegalovirus protease (PDB ID: 1NJU), as it presents 30% of identity with HSV-1 protease with a highly conserved active site. Moreover it presents an inhibitor similar with the benzoxazinones studied herein. The ligand was removed from the enzyme and re-docked keeping the backbone of the peptidomimetic rigid. The comparison of re-docking results with the co-crystallized conformation showed a high success rate, with a root mean square deviation (RMSD) of 1.21 Å. These data supported the hypothesis that the experimental binding mode could be reproduced with accuracy using this protocol.

#### 2.3.2. Docking Analysis

**Figure 2:**
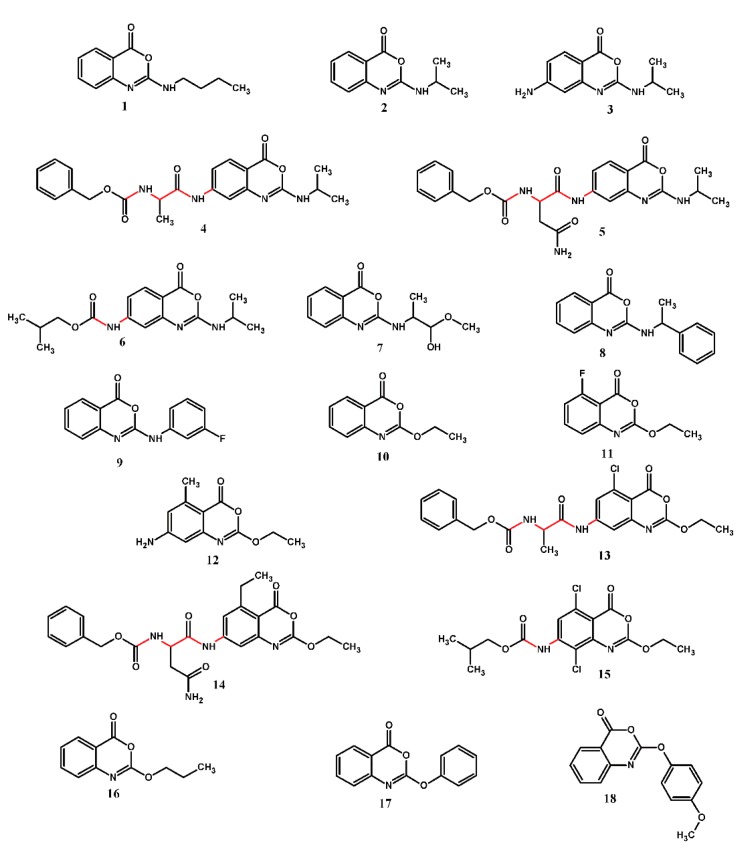
Chemical structures of the benzoxazinone derivative series. Peptidic portions of the backbone (highlighted in red) were treated as rigid bonds.

The benzoxazinones backbone was treated as in validated protocol where the peptidic moiety was kept rigid whereas the non-peptidic moiety and side chains were considered flexible (Figure 2). Each benzoxazinone was docked into the HSV-1 protease active site and their interactions were then analyzed.

**Figure 3:**
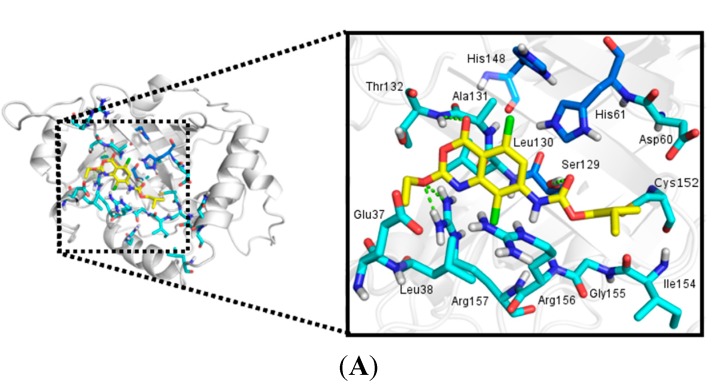

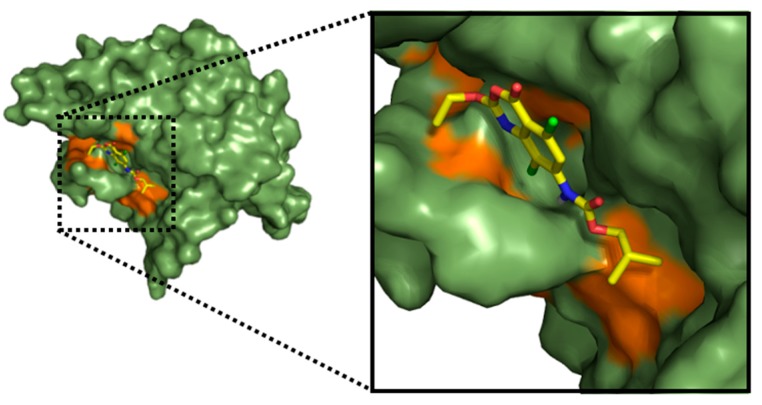
Interactions between benzoxazinone **15** (yellow) and HSV-1 protease residues (blue) observed in the molecular complex. (**A**) Hydrogen bonds (green) involving Ser129 (distance 3.0 Å), Thr132 (distance 3.2 Å), and Arg157 (distances 3.1 and 2.9 Å). Residues in dark blue are members of the catalytic triad; (**B**) Hydrophobic interactions with the residues of the hydrophobic cavity (orange).

The most active benzoxazinone (compound **15**) is involved in four hydrogen bonds with Thr132 (distance of 3.2 Å), Ser129 (distance of 3.0 Å) and Arg157 (distances of 3.1 and 2.9 Å) residues of HSV-1 protease (Figure 3). The importance of Arg157 and Ser129 to the activity of HSV-1 protease has been previously reported [15,19]. The hydrogen bond between benzoxazinone **15** with Ser129, which is a member of catalytic triad and a nucleophile when the enzyme is activated, seems to be one of the most important interactions to the inhibitory activity as this is the only benzoxazinone that interacts with this residue.

Additionally, the hydrogen bond with Arg157 makes this residue less available to stabilize the nucleophilic attack by Ser129, inactivating the enzyme. Compound **15** also presented hydrophobic interactions with Leu38, Leu130, Ala131, Cys152, Ala153, Ile154 residues, contributing to stabilize this inhibitor in the enzyme (Figure 3).

**Figure 4:**
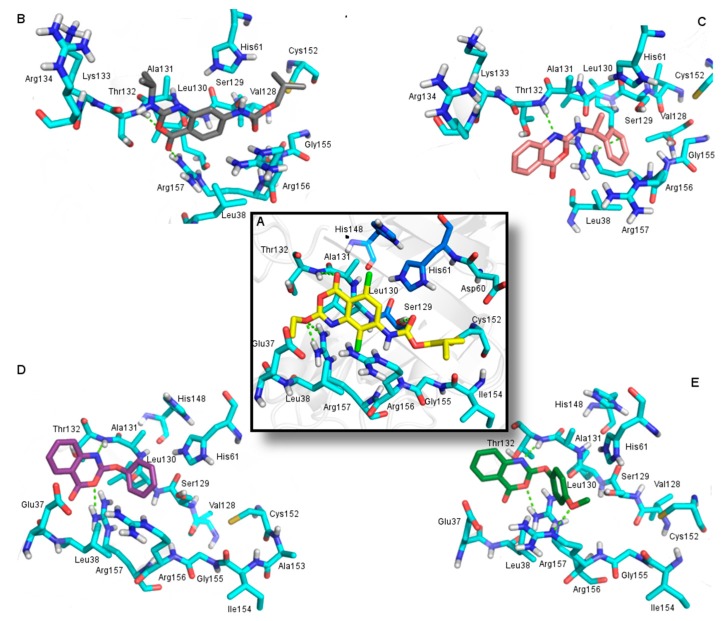
Molecular docking results between HSV-1 protease (blue) and: (**A**) benzoxazinone **15** (yellow); (**B**) benzoxazinone **6** (gray); (**C**) benzoxazinone **8** (pink); (**D**) benzoxazinone **17** (purple) and (**E**) benzoxazinone **18** (green). Hydrogen bonds and cation-π interactions are indicated in green.

Comparison of compounds **15** and **6**, which are structurally similar, but with different inhibitory profile, revealed that compound **6** conserved only two hydrogen bonds with the oxygen of Ser129 and nitrogen of Arg157 (Figure 4).

The absence of the chlorine atom located at the R^3^ position could be related to the poor activity of compound **6**, corroborating the SAR analysis. Comparison of the docking of compounds **18**, **17** and **8** which present similar substituents at the R^1^ position indicated the influence of the number and type of interactions on activity. Compound **18**, the second most active, showed hydrogen bonds with the Thr132 backbone nitrogen and with the side chain nitrogens of the Arg156 and Arg157 residues with distances of 3.4, 2.9 and 3.1 Å, respectively. Compound **17** presented hydrogen bonds with the Thr132 backbone nitrogen (distance of 3.0 Å) and the Arg157 side chain nitrogen (distance of 2.8 Å). Compound **8** is the less active among the three and presented only one hydrogen bond with the Thr132 backbone nitrogen and a cation-π interaction with Arg156 with distances of 3.4 Å and 3.9 Å, respectively. Cation-π interactions present lower energy than hydrogen bonds [22] and may explain the *ca.* 6- and 24-fold difference in activity compared to compounds **17** and **18**, respectively. It is important to note that **18** is the only compound that presented an interaction with Arg157, which is probably directly related to its good activity (Figure 4).

**Figure 5:**
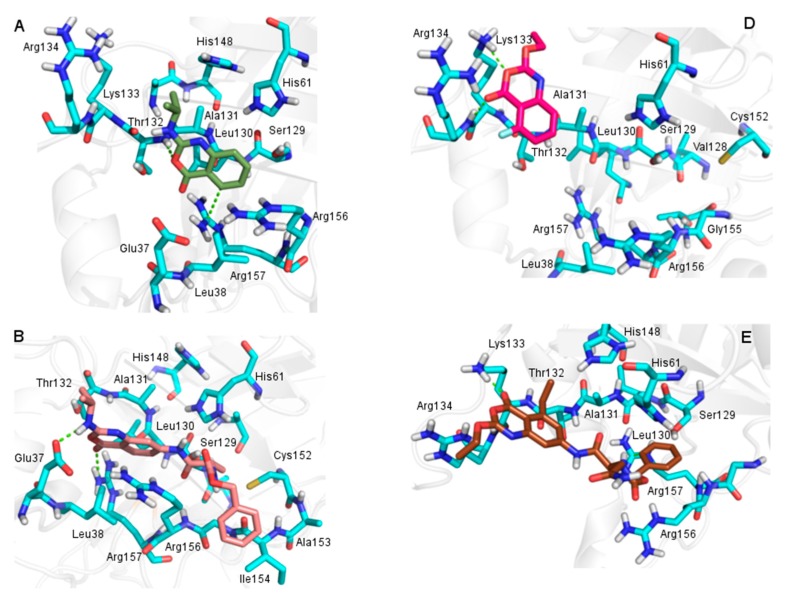

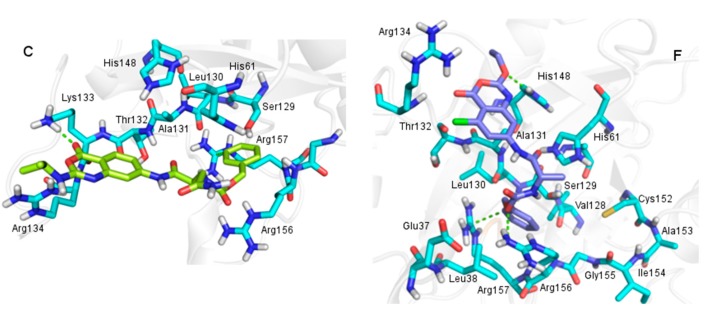
Representation of the molecular docking results between HSV-1 protease (blue) and aminobenzoxazinones **2** (**A**); **4** (**B**) and **5** (**C**) and oxybenzoxazinones **11** (**D**); **14** (**E**) and **13** (**F**). The comparison showed bulky substituents affecting the ligands positioning at binding site. Hydrogen bonds and cation-π interactions are indicated in green.

As can be observed by comparing the docking results of aminobenzoxazinones **2**, **4**, **5** and oxybenzoxazinones **11**, **13**, **14**, bulky substituents at position R^2^ as well as the absence of substituents at this position seem to decrease the activity, reinforcing the SAR analysis (Figure 5). In contrast, the presence of chlorine atom at positions R^3^ and specially R^4^ increased the activity. The presence of this type of substituent triggers the positioning of the compounds away from the Ser129. Nevertheless, compound **13** has shown an activity profile (IC_50_ = 7 µM) probably related to a hydrogen bond with the Arg156 side chain nitrogen with a distance of 3.1 Å. Besides hydrogen bonds, results pointed to the orientation of a chlorine atom towards a hydrophobic cavity as important to increase the activity (Figure 5).

**Figure 6:**
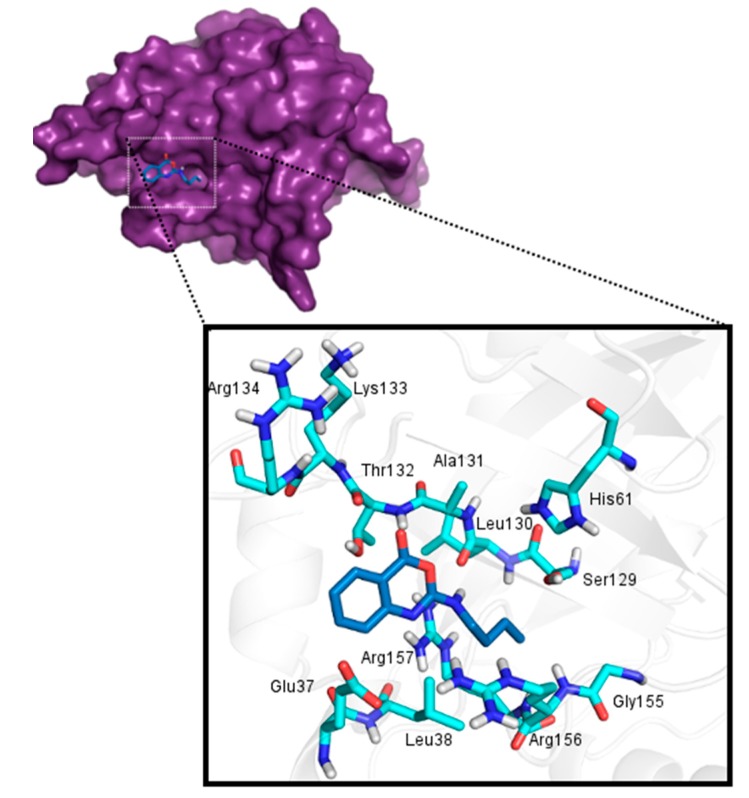
Molecular docking between HSV-1 protease and benzoxazinone 1. The absence of hydrogen interactions and the presence of hydrophobic interactions could be responsible for positioning the ligand at the binding cavity.

Compound **1** presented no hydrogen bonds with HSV-1 protease, which may explain its low activity (Figure 6). However, it was well positioned in the HSV-1 protease catalytic site, probably due to the hydrophobic interaction between the apolar chain and hydrophobic amino acids of the enzyme. The hydrophobic interactions seems to be deeply associated with the positioning of compounds but are not the unique factors to increase activity. We could infer that hydrogen bond interactions are oriented by hydrophobic interactions of the R^2^ substituents.

The benzoxazinone **15** showed a particular binding mode with HSV-1 protease, characterized by the presence of a hydrogen bond with Ser129. This evidence supports the hypothesis that the mentioned interaction could facilitate the approximation of the nucleophile Ser129 to the carbonyl. The proximity of the atoms consequently could lead to the reaction for covalent bond formation, which would be in accordance with the proposed mechanism of action [18].

**Figure 7:**
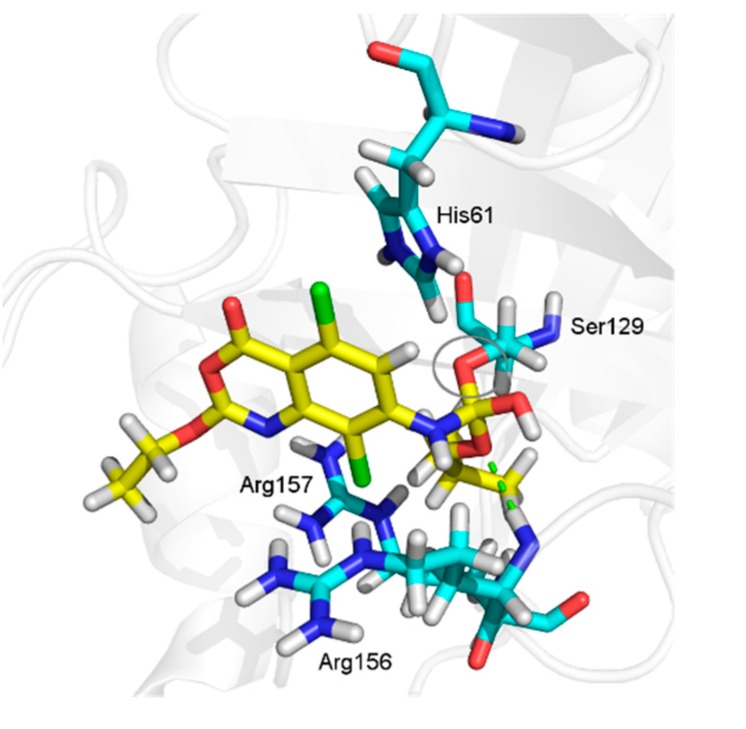
Covalent docking between benzoxazinone **15** and HSV-1 protease, showing the presence of a covalent bond between Ser129 and the R_2_ substituent of the ligand.

To evaluate this hypothesis, we also performed the covalent docking between benzoxazinone **15** and HSV-1 protease. As expected, the results showed the presence of the covalent bond between the Ser129 side chain and the benzoxazinone (Figure 7). Other interactions were also observed as a cation-π and a hydrogen bond with Arg156 with a distance of 3.1 Å (Figure 7) [15,19].

### 2.4. In Silico Analysis of Pharmacokinetic Properties

**Figure 8:**
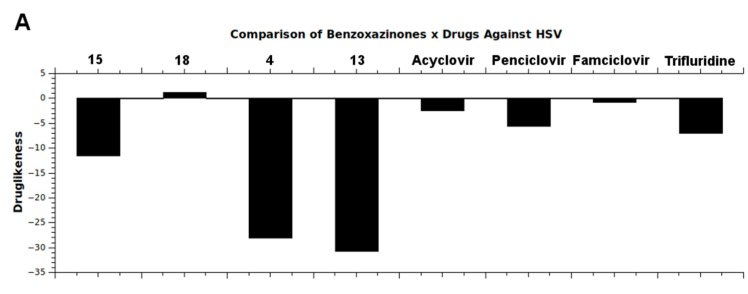

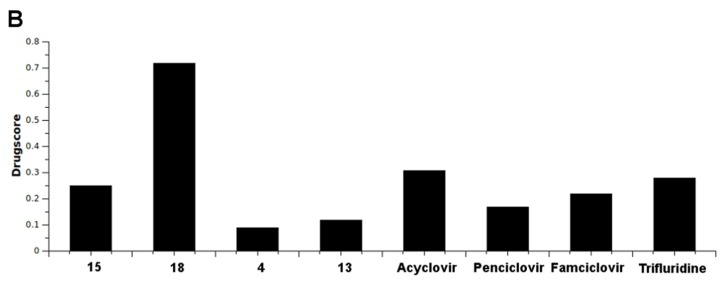
Results obtained from (**A**) druglikeness and (**B**) drugscore calculations. Comparison between benzoxazinones and commercial drugs against HSV shows better results for benzoxazinone **18**.

Druglikeness and drugscore studies were carried out to compare the most actives benzoxazinones (**4**, **13**, **15**, **18**) with some currently available drugs against HSV including acyclovir, penciclovir, famciclovir and trifluridine. It is important to highlight that all benzoxazinones fulfilled Lipinski’s rule of five, and therefore theoretically presented good oral bioavaliability [23]. Although the benzoxazinone **15** was the most active compound, the best druglikeness and drugscore results were achieved for compound **18** that was also better than the commercial drugs (Figure 8). The druglikeness evaluation revealed a positive value only for compound **18** in addition to the higher drugscore for this compound.

**Figure 9:**
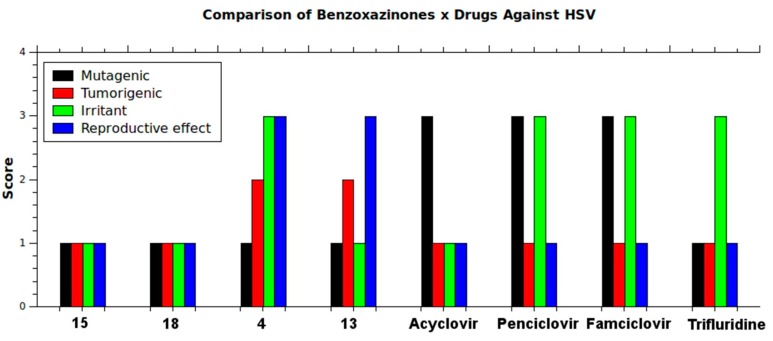
Results obtained from toxicity risks assessment wherein scores are given as 1 (Low Risk), 2 (Medium Risk), or 3 (High Risk). Comparison between benzoxazinones and commercial drugs against HSV shows better results for benzoxazinones **15** and **18**.

We also performed toxicity risks analysis using Osiris Property Explorer. Compounds **15** and **18** showed better mutagenicity, tumorigenicity, irritant and reproductive effect results than the commercial drugs (Figure 9). Taken together these results suggest that **18** could be an interesting lead compound for further studies and structural modifications.

## 3. Experimental Section

### 3.1. Structure-Activity Relationship

We constructed the three-dimensional structures of 18 benzoxazinone HSV-1 protease inhibitors previously described by Jarvest and coworkers (Table 1) [18]. All calculations were performed using SPARTAN’10 (Wavefunction Inc. Irvine, CA, USA). The structures were minimized and a conformational analysis was done using Molecular Mechanics Force Field (MMFF) [24]. The equilibrium geometry was obtained in vacuum using a semi-empirical RM1 module. To obtain the electronic properties of the RM1 minimal energy conformation of each benzoxazinone, they were submitted to a Density Functional Theory (DFT) B3LYP calculation with a 6-31G* basis set. The electronic (HOMO and LUMO energy, electrostatic potential and dipole moment) and structural properties (molecular weight, area, number of hydrogen bonds donors and acceptors) were calculated for all compounds.

### 3.2. Comparative Modeling

Since there is no crystal structure of HSV-1 protease currently available, we built a three-dimensional structure employing comparative modeling. The amino acid sequence was retrieved from the Uniprot database [25] (access number Q69087). BLAST-P [26] was used to identify homologous structures by searching the structural database of protein sequences in the Protein Data Bank. Based on the highest percentage of sequence identity (71.7%) the crystal structure of HSV-2 protease was selected as structural template (PDB ID: 1AT3, 2.5 Å resolution) [11]. Models were generated using two different methods: (a) through SWISS-MODEL server on the automatic mode [27] and (b) performing the alignment with T-Coffee [28] server followed by model construction using Modeller program [29,30,31,32,33,34,35]. The overall stereochemical quality of the developed models was assessed by using PROCHECK [36,37,38,39] and the environment profile was checked using Verify-3D [40,41]. All validations were performed within SAVES server.

### 3.3. Molecular Docking

In order to investigate the binding mode of a series of benzoxazinones we performed molecular docking studies. The AutoDock 4.2 program was used [42] running on a Windows-based PC. First, the 3D structures of all benzoxazinones were built as described in the SAR methodology. The ligands were partially flexibly docked to HSV-1 and the docking files were prepared using AutoDockTools (ADT). The protein was treated as rigid, polar hydrogen atoms were added, nonpolar hydrogen atoms were merged and Gasteiger charges were assigned by default. The grid map dimensions were 36 × 40 × 60 points with 0.375 Å spacing and the coordinates to centralization were defined by validation step. Docking studies were carried out using the empirical free energy function and the Lamarckian Genetic Algorithm (LGA). The default parameters of LGA modified for calculations were elitism (15), crossover rate (0.6), number of energy evaluations (25,000,000) and population size (150). Results were analyzed using PyMol program (The PyMOL Molecular Graphics System, Version 1.3, Schrödinger, LLC, San Francisco, CA, USA).

We also performed a covalent docking between the most active benzoxazinone 15 and the HSV-1 protease, with the software GOLD Suite [43,44,45,46]. The molecular docking started setting the default parameters for Serine Proteases, following the binding cavity centralization in the side chain oxygen of Ser 129. The dimension of 15 radius was selected to the binding cavity. The GOLD fitness function used was Chemscore, allowing the rescore by Goldscore. A total of 50 solutions were obtained and the 5 best solutions were analyzed using PyMol program.

### 3.4. In Silico Pharmacokinetic Properties

There are many approaches that assess the druglikeness of compounds based on topological descriptors, fingerprints of molecular druglikeness, drugscore, structural keys or other properties as clogP and molecular weights. In this work we used the Osiris program [47] to calculate, for the most active compounds, the toxicity risks and the fragment-based druglikeness and drugscore, which combines the molecular properties above.

## 4. Conclusions

In this work we present the construction and validation of the HSV-1 protease structure and a molecular docking study between this enzyme and eighteen previously tested benzoxazinones. The results confirmed the importance of some residues including Arg156, Arg157 and mainly Ser129 for protease activity and of hydrophobic interactions on ligand anchoring, not yet described. The involvement of Ser129 in an exclusive hydrogen bond with benzoxazinone **15**, the most active of the series, reinforced the importance of this residue for activity. Although compound **15** was the most active, druglikeness and drugscore studies revealed compound **18** to have the best overall profile. These findings could help in drug design and indicate compound **18** as a potential hit in the search for new drugs against HSV-1.
